# AQP9 (Aquaporin 9) Determines Arsenic Uptake and Tolerance in Human Hepatocellular Carcinoma Cells In Vitro

**DOI:** 10.7759/cureus.26753

**Published:** 2022-07-11

**Authors:** Dingyan Yao, Sishi Liu, Fuzhi Lian, Xianrong Xu, Jun Yang, Rong Chen, Yifei Cao

**Affiliations:** 1 School of Public Health, Hangzhou Normal University, Hangzhou, CHN

**Keywords:** phosphorylation, p38, aqp9, hepg2, arsenic tolerance

## Abstract

Arsenic-based therapeutic strategies, even though promising for acute promyelocytic leukemia (APL), are limited by arsenic-related toxic effect and resistance with unknown mechanisms. The purpose of this study is to better understand the different sensitivities of hepatocellular carcinoma cells to arsenic and its mechanism. Arsenic-sensitive liver cancer cell line (HepG2) and arsenic-resistant HepG2 (AsHepG2) cells are employed to study the role of aquaporin 9 (AQP9) in arsenic uptake and tolerance. The half-maximal inhibitory concentration (IC_50_) value of arsenic in AsHepG2 cells (15.59 ± 1.36 µM) is significantly higher than that in HepG2 cells (7.33 ± 0.93 µM; p= 0.0288). We demonstrated that, with the treatment of sodium arsenite (NaAsO_2_), arsenic was accumulated at a significantly lower level in AsHepG2 cells in comparison with HepG2 cells (p= 0.00549). Further, arsenic level in AsHepG2 cells reaches a plateau after six hours of treatment, whereas arsenic continues to increase in HepG2 cells during the entire experimental period. Mechanistic study showed that the expression of AQP9 is decreased in a dose-dependent manner in AsHepG2 cells, but no significant difference in HepG2 cells. Furthermore, NaAsO_2 _dramatically increases AQP9 and p38 phosphorylation, which may partially regulate arsenic sensitivity in both cell lines. In conclusion, the expression and phosphorylation of AQP9 regulated by p38 kinase are involved in the arsenic uptake, thus regulating cellular arsenic sensitivity.

## Introduction

Arsenic is widely granted as chemotherapeutic agents to treat certain types of diseases [1], including acute promyelocytic leukemia (APL) [2]. Despite its effectiveness, the resistance against arsenic chemotherapy was observed in many cancer types like leukemia and lung cancer, as well as cell lines such as Chinese hamster ovary (CHO) cells [3,4]. Such resistance or tolerance limited its applications in cancer therapy; thus, elucidation of the underlying resistance mechanisms would be helpful in developing arsenic-based effective therapeutic strategies.

Some studies suggested that acquired tolerance to arsenic is mainly due to the changes in cellular uptake and efflux pathways. For example, aquaporin (AQP) water channels, 13 members of the aquaglyceroporin family, are considered the principal entry routes for As(III) in bacteria [5,6], Leishmania protozoa [7], yeast [8], and vertebrate [9,10]. Among these, AQP9 plays a particularly important role in arsenic uptake [9,11-13]. In yeast *Saccharomyces cerevisiae*, the AQP9 homolog Fps1p contributes to show high sensitivity to both As(III) and trivalent antimony Sb(III) [14]. Moreover, AQP9 overexpression can restore arsenic sensitivity in *S. cerevisiae* in which the gene FPS1 was deleted [8]. AQP9 can also mediate intracellular arsenic accumulation and arsenic hypersensitivity in leukemia cell line K562, human lung cancer cells, liver cancer cell line (HepG2) [15,16], and primary mouse hepatocytes [17]. Our previous studies also showed that AQP9 may play an essential role in regulating arsenic influx [18].

It is noted that As(III) can target p38 kinase, which then initiates several stress response genes [19-21]. In contrast, p38 inhibition can suppress the expression of water channel protein aquaporin 4 (AQP4) and aquaporin 9 (AQP9) in cultured rat astrocytes [22]; thus, it can enhance arsenic trioxide (As_2_O_3_)-induced cytotoxicity in myeloma cells [23]. Studies from our laboratory and collaborators’ laboratory found that the downregulation of mitogen-activated protein kinase (MAPK)-p38 pathway may increase the uptake of arsenic to sensitize cells through regulating AQP9 [24]. However, whether p38 regulates AQP9 activity, thus regulating arsenic uptake and/or sensitivity, needs to be defined. Therefore, we intended to study arsenic uptake by two opposing cell lines: the arsenic-sensitive liver cancer cell line (HepG2) and its modified counterpart arsenic-resistant HepG2 (AsHepG2). We found that AQP9 is involved in arsenic uptake in hepatocellular carcinoma cells, in which p38-MAPK may play a limited role. This article was previously posted to the Research Square preprint server on June 9, 2021.

## Materials and methods

Cell lines

HepG2 is one human hepatocellular carcinoma-derived cell line, obtained from the cell bank of the Chinese Academy of Sciences in Shanghai, China. Arsenic-resistant HepG2 (AsHepG2) cells were generated by continuously treating HepG2 cells with a low concentration of arsenic [18,25].

Cell viability assay

Cell viability was determined with Cell Counting Kit-8 (CCK8, Beyotime, China). Briefly, 104 cells/100 µl seeded on a 96-well plate were treated with various doses of sodium arsenite (NaAsO_2_) including 0, 4, 8, 16, 32, and 64 µmol/l for 24 hours. The CCK8 reagents were added accordingly to the kit instruction for one hour and then subjected to absorbance at 450 nm by a microplate reader (Bio-Rad, Hercules, CA, USA).

Measurement of arsenic accumulation

Cells were washed three times with phosphate-buffered saline (PBS) and harvested in 0.5 ml nitrosonitric acid (98%). Arsenic accumulation was measured using an inductively coupled plasma mass spectrometer (ICP-MS, HP4500; Yokogawa Analytical Systems, Japan).

Quantitative polymerase chain reaction (qPCR) 

Total RNA extraction and complementary DNA (cDNA) synthesis were based on the description from our lab, followed by real-time polymerase chain reaction (PCR) with these specific primers: AQP9 (forward 5′-ACT CAG TGT CAT CAT GTA GTG G-3′ and reverse 5′-CAC CTC AGG CTT ACA AGA ACA-3′; β-actin (functions as internal control, forward 5′-TGG CAC CCA GCA ATG AA-3′ and reverse 5′-CTA AGT CAT AGT CCA CCT AGA AGC A-3′), on a GeneAmp PCR® system 7300 (Applied Biosystems, Carlsbad, CA, USA) using SYBR method (Takara Bio Inc., Kusatsu, Shiga, Japan). Measurement of each sample was independently repeated three times; the relative mRNA expression was analyzed by the delta Ct (ΔCt) method; and data were expressed as 2−ΔΔCt, where Ct values were analyzed according to a previously reported method [26].

Immunoprecipitation and Western blot analysis

Immunoprecipitation and Western blot were performed based on the description from our lab previously. Cells were lysed, separated, and transferred to a polyvinylidene difluoride (PVDF) membrane. The membranes were subjected sequentially to blocking buffer, washing buffer, and monoclonal antibodies against AQP9 (1:500, Santa Cruz Biotechnology, Santa Cruz, CA, USA), p38 (1:500, Abcam, London, UK), or a rabbit polyclonal antibody to phospho-p38-MAPK (Thr180/Tyr182; 1:500, Abcam). β-actin (1:500, Santa Cruz Biotechnology) served as a loading control. Then the reaction was incubated with horseradish peroxidase-conjugated secondary antibodies (HRP) and pictured with an enhanced chemiluminescence system (Bio-Rad).

p38 inhibition assay

To evaluate the effects of p38 activation on the level of AQP9 phosphorylation, cells pretreated with or without the p38 inhibitor SB203580 (18 μM; Beyotime, Shanghai, China) for 30 minutes were treated with 12 μM NaAsO_2_ for two hours. Cell lysates were extracted using radioimmunoprecipitation assay (RIPA) buffer and subjected to sodium dodecyl sulfate-polyacrylamide gel electrophoresis (SDS-PAGE) and Western blot as described above.

Data analysis

Data were expressed as mean ± SE. One-way analysis of variance (ANOVA) was used with Tukey’s multiple comparison test to compare differences in means among treatment groups. Statistical Package for the Social Sciences (SPSS) 16.0 software (SPSS, Chicago, IL, USA) was used for the statistical analysis. Differences with probability (P) values < 0.05 were considered significant.

## Results

AsHepG2 is more resistant to arsenic than HepG2

**Figure 1:**
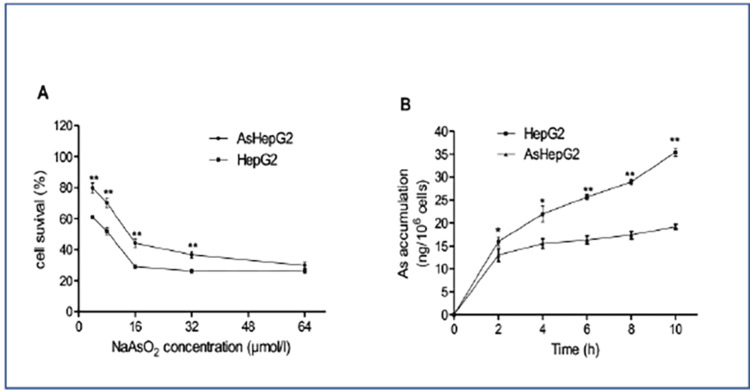
Cell viability and arsenic accumulation in arsenic-resistant liver cancer cell line (AsHepG2) and HepG2 cells with sodium arsenite (NaAsO2) treatment. Cell viability and arsenic accumulation in AsHepG2 and HepG2 cells with NaAsO_2_ treatment. (A) Cells grown in a 96-well plate were treated with different concentrations of NaAsO_2_ (4, 8, 16, 32, and 64 µmol/l) for 24 hours, and Cell Counting Kit-8 (CCK8) assays were performed. Results are expressed as cell survival % and represent mean ± SEM of five determinations. * p = 0.0288, ** p = 0.00549. (B) Cells were treated with 16 µmol/l NaAsO_2_ for 24 hours, and arsenic accumulation was determined by inductively coupled plasma mass spectrometer (ICP-MS) methods. Results are expressed as arsenic content (ng) per million cells and represent mean ± SEM (n = 3). * p = 0.0182, ** p = 0.000241.

The viability of HepG2 and AsHepG2 was quantified by half-maximal inhibitory concentration (IC_50_) of NaAsO_2_. The IC50 in AsHepG2 (15.59 ± 1.36 μM) is statistically enhanced, compared to HepG2 (7.33 ± 0.93 μM) (p = 0.0288) (Figure 1A). Treatment of 16 μM of NaAsO_2_ showed that arsenic in AsHepG2 was reduced compared to HepG2 cells (p < 0.00549; Figure 1B), suggesting that reduced arsenic can lead to increased tolerance.

AQP9 regulates intracellular arsenic accumulation 

**Figure 2:**
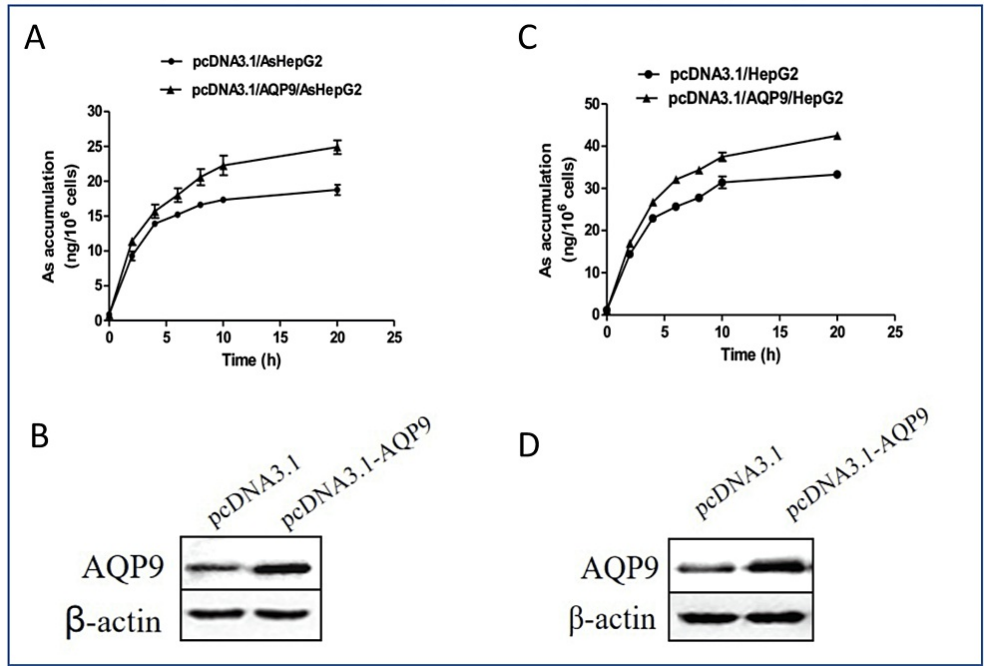
Effects of aquaporin 9 (AQP9) on As accumulation in arsenic-resistant liver cancer cell line (AsHepG2) and HepG2 cells. AQP9 expression in AsHepG2 and HepG2 cells with sodium arsenite (NaAsO_2_) treatment. (A) AQP9- or empty vector-transfected AsHepG2 cells were exposed to NaAsO_2_ (16 µmol/l) for two, four, six, eight, 10, and 20 hours; arsenic accumulation was determined by inductively coupled plasma mass spectrometer (ICP-MS). Data presented are mean ± SE (n = 3). (B) Western blotting of molecule AQP9 was performed with 20 µg of AsHepG2 cells lysates, β-actin functions as an internal control. (C) AQP9- or empty vector-transfected HepG2 cells were exposed to NaAsO_2_ (16 µmol/l) for two, four, six, eight, 10, and 20 hours; arsenic accumulation was determined by ICP-MS. Data presented are mean ± SE (n = 3). (D) Western blotting of molecule AQP9 was performed with 20 µg of HepG2 cells lysates, β-actin functions as an internal control.

AQP9 was shown to increase the uptake and toxicity of arsenic in lung cancer and leukemia cells [15,27]. Similarly, overexpression of AQP9 in both AsHepG2 (Figures 2A, 2B) and HepG2 (Figures 2C, 2D) cells by pcDNA3-AQP9 resulted in enhanced intracellular arsenic than control cells (Figures 2A-2D). In AQP9-overexpressed AsHepG2 cells, a linear relationship between arsenic quantity and treatment period was noticed only during the first 10 hours of time, and then the rate of uptake dramatically slows down during 10-20 hours (Figures 2A, 2B). In contrast, intracellular arsenic gradually accumulates during the course of the experiment in AQP9-overexpressed HepG2 cells (Figures 2C, 2D).

The expression of AQP9 is compromised with NaAsO_2_ in AsHepG2 cells

**Figure 3:**
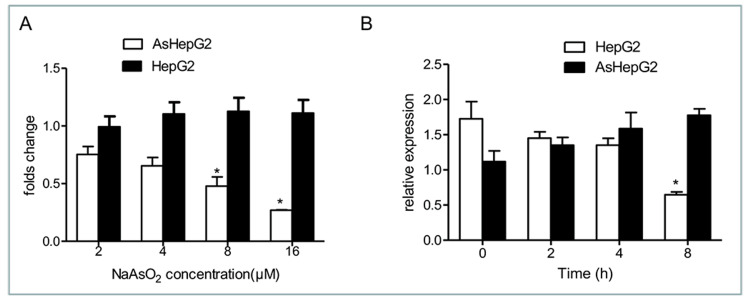
Effect of sodium arsenite (NaAsO2) on aquaporin 9 (AQP9) mRNA levels in arsenic-resistant liver cancer cell line (AsHepG2) and HepG2 cells. (A) Cells were treated with 2, 4, 8, or 16 µmol/l NaAsO_2_ for two hours. (B) Cells were treated with 12 µmol/l NaAsO_2_ for two, four, and eight hours. Total RNA was isolated by Trizol, and a real-time polymerase chain reaction (RT-PCR) was performed. Data presented are mean ± SE (n = 3). * p = 0.015, 0.025, 0.011.

Given the fact that AQP9 regulates arsenic accumulation, we examined whether exogenous arsenic alters the endogenous AQP9 expression. Our results showed that NaAsO_2_ dramatically decreased AQP9 mRNA in time- and dose-dependent manners in AsHepG2 cells (p = 0.015), but not in HepG2 cells (Figures 3A, 3B). Together, under conditions of acute toxicity, compromised AQP9 may attribute to tolerance.

NaAsO_2_ regulates AQP9 activity in non-modified liver cancer cells

**Figure 4:**
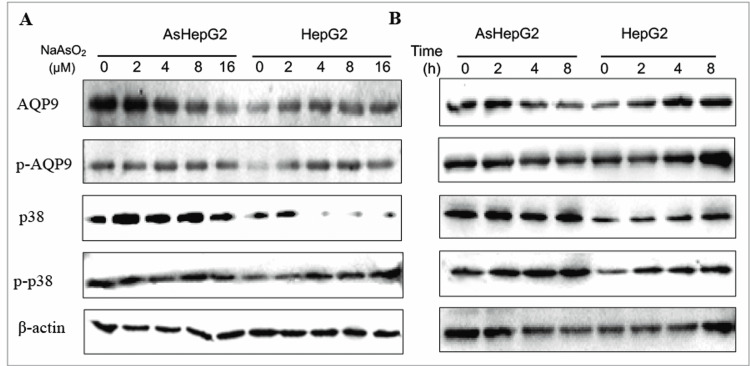
Regulation of sodium arsenite (NaAsO2) on aquaporin 9 (AQP9) and p38 in arsenic-resistant liver cancer cell line (AsHepG2) and HepG2 cells. For dosage manner (A), cells were treated with 0, 2, 4, 8, and 16 µmol/l NaAsO_2_ for two hours, and Western blotting was performed with 20 µg of cell lysates. For the time course (B), cells were treated with 16 µmol/l NaAsO_2_ for zero, two, four, and eight hours. Data presented are mean ± SE (n = 3). p-AQP9: phospho-AQP9, p-p38: phospho-p38.

To further examine arsenic tolerance, we analyzed the function of NaAsO_2_ on AQP9 expression and phosphorylation status. NaAsO_2_ decreased AQP9 expression (Figures 4A, 4B) in AsHepG2 in dose- and time-dependent manners; on the contrary, no change at the protein level was noticed in HepG2 cells (Figure 4B). AQP9 activity alteration was not noticed during NaAsO_2_ treatment in arsenic-resistant AsHepG2. But, in arsenic-sensitive HepG2 cells, NaAsO_2_ dramatically stimulated AQP9 phosphorylation (Figure 4A). As a result, AQP9 activity may affect intracellular arsenic uptake only in non-modified HepG2.

p38 may regulate AQP9 activity

**Figure 5:**
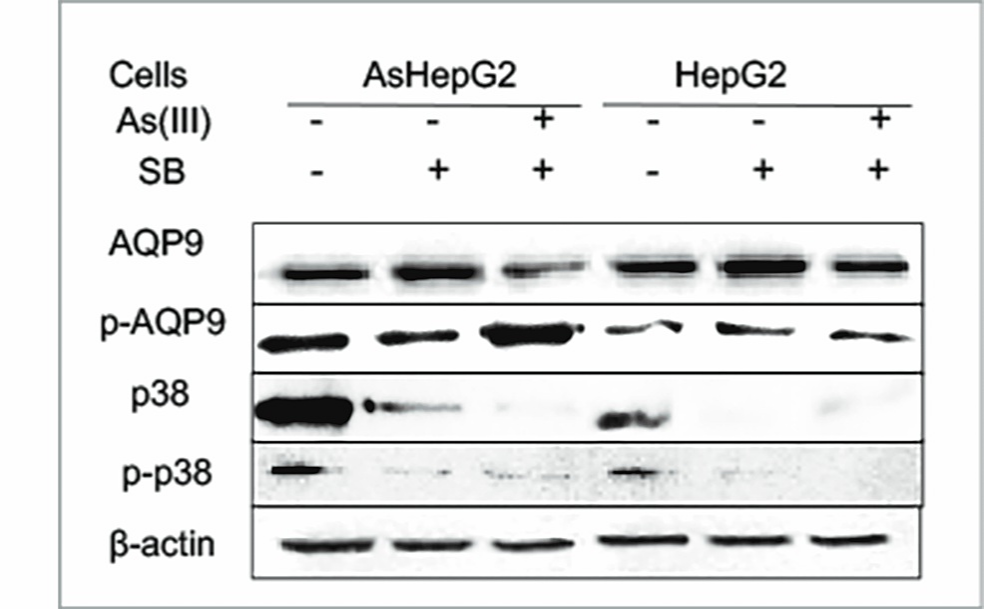
Effect of sodium arsenite (NaAsO2) and SB203580, a p38 mitogen-activated protein kinase (MAPK)-specific inhibitor, on the protein and phosphorylation levels of aquaporin 9 (AQP9) in arsenic-resistant liver cancer cell line (AsHepG2) and HepG2 cells. Cells were treated with 16 µmol/l NaAsO_2_ for two hours after incubation with or without SB203580 (18 µmol/l) for 30 minutes, and Western blotting was performed using 20 µg of cell lysates. Protein and phosphorylation levels of AQP9 and p38 were detected with anti-AQP9, anti-p38, and anti-phospho-p38 (p-p38) antibody, respectively. Phosphorylated AQP9 (p-AQP9) was determined by immunoprecipitation using anti-phosphoserine antibody. β-actin functions as an internal control. Data presented are mean ± SE (n = 3).

Fps1p (AQP9 homolog in yeast) could be stimulated by Hog1p-a p38 counterpart, and then it decreases arsenic uptake [8]. As shown in Figure 4, p38 expression was significantly different with NaAsO_2_ treatment in AsHepG2 cells, but not in HepG2 cells. However, p38 activity was enhanced in dose- and time-dependent manners with NaAsO_2_ treatment (Figures 4A, 4B), implying that p38 may regulate AQP9 activity. In parallel, the involvement of p38 in AQP9 activity was investigated by p38 blocker SB203580, with obvious decreases in AQP9 expression, but AQP9 phosphorylation was not affected significantly (Figure 5), indicating that p38 may only partially regulate AQP9 phosphorylation.

## Discussion

In our current study, to our best knowledge, we found that AQP9 functions as a checkpoint for arsenic uptake and tolerance by two distinguished hepatocyte cell lines, arsenic-sensitive HepG2 and its counterpart arsenic-resistant AsHepG2. Arsenic accumulation through AQP9 has been focused on recently, such as in yeast or mammalian cell lines [8,15,17,27]. For example, Fps1p (one AQP9 homolog in *S. cerevisiae*) facilitates the high sensitivity to arsenic uptakes [14], and the mammalian-derived AQP9 can restore arsenic sensitivity in an *S. cerevisiae* with FPS1 deficiency [8]. It has been reported that AQP9 is involved in arsenic uptake in K562, HepG2 [28], and human umbilical vein endothelial cells (HUVEC) from our laboratory [18]. 

To confirm the function of AQP9 in arsenic uptake, we used liver cancer cell lines as in vitro models. Data from AsHepG2 revealed a better survival trend and lower arsenic toxicity than HepG2 cells, and in parallel, R15 cells harbored lower arsenic than its parent counterpart lung cancer cell line CL3 [29]. Similarly, the accumulation of arsenic in arsenic-resistant cells was lower than that in ECV-304 cells [18]. These data confirmed that decreased arsenic accumulation through restriction of arsenic uptake is one of the mechanisms underlying arsenic tolerance.

AQP9 is a water-glycerol channel that allows extracellular arsenic to move into cells, thereby leading to intracellular accumulation and cytotoxicity, which may imply the phenomena in hepatocytes in our current study. In physiologic conditions such as in primary cultured chorion cells, different sensitivities to arsenic due to different AQP9 expression were noticed [30]. Different groups have shown that AQP9 facilitates cellular sensitivity to arsenic therapy against some malignancies. For example, NB4 (one APL cell line) expresses the highest level of AQP9 and is most sensitive to As_2_O_3_, with the lowest IC_50_ value for arsenic among leukemia cell lines. In contrast, the chronic myeloid leukemia (CML) cell K562 has very low endogenous AQP9 and thus harbors the least sensitivity or efficacy to As_2_O_3_ treatment [15]. However, AQP9 overexpression can dramatically sensitize the K562 cells to As_2_O_3_ [15]. 

In our study, different from AsHepG2, AQP9 was phosphorylated in HepG2 in dose- and time-dependent manners, which are in concert with arsenic uptake in these cells (Figure 4). Thus, AQP9 phosphorylation could regulate arsenic influx, similar to our previous study in another model [18]. AQP9 expression could be regulated by p38-MAPK signaling pathway, for example, it was reported that p38 regulated AQP expression in *S. cerevisiae* [8,22]. Our experiments saw the enhanced p38 expression and phosphorylation in HepG2, compared to AsHepG2 with arsenic treatment, implying that p38 may, at least in part, increase AQP9 function. Also, p38 blocker SB203580 facilitates AQP9 function in AsHepG2 cells (Figure 5), further suggesting that p38 can partially affect AQP9 function. Taking all the results of this study into consideration, we propose that many kinases could contribute to AQP9 function, p38 homolog, and yeast Hog1p and can stimulate AQP9 homolog [8]. Identification of the kinase(s) that can phosphorylate and facilitate channel AQP9 will be our next focus.

## Conclusions

In this study, attributable to two distinguished hepatocyte cell lines, arsenic-sensitive HepG2 cells and its modified version arsenic-resistant HepG2 (AsHepG2) cells, we found that molecules AQP9 and p38 are crucial in arsenic uptake. The results provide primary mechanisms underlying arsenic tolerance or detoxification in hepatocytes, which encourage us to focus on the potential of AQP9 as one target against arsenic.
